# Expanding the Phenotype of PARK‐ *PRKN* to Spastic Paraplegia: A Report of Two Cases

**DOI:** 10.1002/mdc3.70662

**Published:** 2026-04-30

**Authors:** Nicolas Geoffre, Thomas Ollivier, Guillaume Baille, Luc Defebvre, Vincent Huin, Eugenie Mutez

**Affiliations:** ^1^ Univ. Lille, Inserm, CHU Lille, U1172‐LilNCog‐Lille Neurosciences & Cognition Lille France; ^2^ Department of Toxicology and Genopathies Lille University Hospital Lille France; ^3^ Neurology and Movement Disorders Department, Expert Center for Parkinson's Disease Lille University Hospital Lille France; ^4^ Neurology Department Foundation Rothschild Paris France

**Keywords:** early‐onset Parkinson's disease, gait disorders, genetic disorder, hereditary spastic paraplegia, *PRKN*

Biallelic pathogenic variants in *PRKN* are the most common genetic cause of autosomal recessive early‐onset PD (EOPD).^1^, ^2^ We report two patients initially presenting with spastic paraplegia and subsequently diagnosed with PARK‐*PRKN*.

A 41‐year‐old (yo) Caucasian woman presented with gait impairment, lower‐limb pain, and urinary urgency since age 38. Her sibling was diagnosed with PD at age 41. Clinical examination revealed a subtle rest tremor, spastic paraparesis (hyperreflexia, extensor plantar response, ankle clonus). Treatment included physiotherapy and baclofen. Vitamin, inflammatory, metabolic, and infectious tests, brain and spine MRI, lumbar puncture analyses, motor and sensory evoked potentials and neuro‐ophthalmologic examination were normal. After 3 years, she could walk a few hundred meters, and had bilateral tremor. DATScan® revealed asymmetrical striatal denervation. A levodopa challenge test showed 41% responsiveness, supporting the diagnosis of PD. She was treated with rasagiline, pramipexole and botulinum toxin injections. Initial genetic testing (MLPA and Sanger sequencing) identified a compound heterozygosity in *PRKN* associating a deletion of the exons 3 to 6 and a likely pathogenic variant: NM_004562.3:c.1298A>C, p.(His433Pro) (Fig. 1A, Supplementary Data). Subsequent whole exome sequencing retrieved no other related pathogenic variants. At 51, the disease had progressed very slowly and was controlled by treatment (MDS‐UPDRS‐III score 8/132 on medication). The patient remained without levodopa therapy because of disabling dyskinesia after each initiation (Video S1).

**Figure 1:**
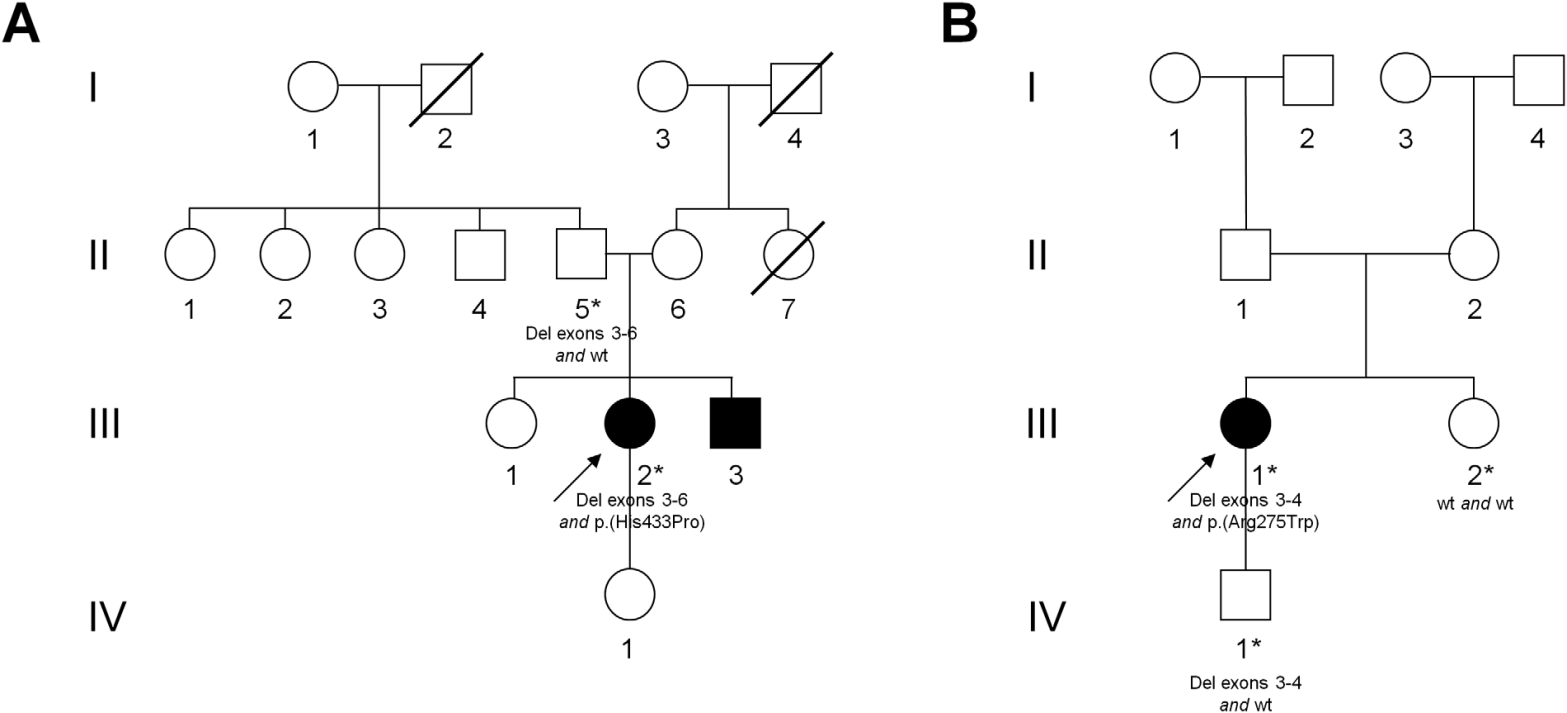
Pedigrees and *PRKN* variants of the two families. Circles denote women. Squares denote men. Probands are indicated by an arrow. Filled black symbols denote clinically affected members. Open symbols indicate unaffected individuals. A forward slash indicates deceased individuals. *Genotyped individuals; Del, deletion; wt, wild‐type.

A 38 yo Caucasian woman was referred for a bilateral hand resting tremor. She had since age 20 an unexplored spastic syndrome that required four spasticity surgeries. Tremor appeared at age 28. Clinical examination confirmed a slow gait, a pyramidal syndrome with global hyperreflexia, spasticity and global akinetic‐rigid syndrome. Brain and medullar MRI, motor and sensory evoked potential, lumbar puncture analyses, and neuro‐ophthalmological examination returned normal. Although a poor levodopa‐challenge response, she reported a long‐term improvement with amantadine, trihexyphenidyl and piribedil alongside physiotherapy. At age 49, DATScan® showed a bilateral dopaminergic denervation. Similar genetic testing revealed a compound heterozygosity in *PRKN*: Deletion of the exons 3–4 and a variant: c.823C>T, p.(Arg275Trp) (Fig. 1B, Supplementary Data). These two variants are the most frequent pathogenic variants.^3^ Subsequent whole exome sequencing retrieved no other pathogenic variants. At age 63, parkinsonism had progressed very slowly (MDS‐UPDRS III 23/132 with rasagiline, without levodopa) (Video S2).

Although hyperreflexia is a well‐known feature of the *PRKN* phenotype,^2^, ^4^ only one study has reported a PARK‐*PRKN* patient with EOPD and spastic paraplegia.^5^ Schneider et al, used transcranial magnetic stimulation (TMS) to evidence corticospinal tract involvement in PARK‐*PRKN* patients.^6^ They hypothesized that this impairment might be a compensatory mechanism from the motor cortex in adaptation to the dopaminergic dysfunction. To test this hypothesis, further studies including TMS or functional imaging could be of interest in large cohort of PARK‐*PRKN* patients. For clinicians, these observations encourage consideration of this diagnosis at the first signs of parkinsonism in patients with spastic paraplegia. From a laboratory perspective, it promotes careful analysis of *PRKN* in such condition.

## Author Roles

(1) Research project: A. Conception, B. Organization, C. Execution; (2) Statistical Analysis: A. Design, B. Execution, C. Review and Critique; (3) Manuscript: A. Writing of the first draft, B. Review and Critique; (4) A. Funding acquisition.

N.G.: 1C, 3A.

T.O.: 1C, 3B.

G.B.: 1C, 3B.

L.D.: 3B.

V.H.: 1A, 1B, 1C, 3A, 4A.

E.M.: 1A, 1B, 1C, 3A, 4A.

All authors approved the submitted version.

## Disclosures

**Ethical Compliance Statement:** The study adhered to the declaration of Helsinki, with written informed consent obtained for genetic testing. Patients gave written informed consent for their personal or clinical details and videos to be published in this study. We confirm that we have read the Journal's position on issues involved in the ethical publication and affirm that this work is consistent with those guidelines. We also confirm patients gave written informed consent for their personal and clinical details to be published in this study.

**Funding Sources and Conflict of Interest:** This work was supported by the University of Lille and the Lille university Hospital (CHU Lille). VH received the following grants: “France Alzheimer,” “France Parkinson” and the “Association des Aidants et Malades à Corps de Lewy” (A2MCL) charities. This work was also funded by grants from the investissements d'avenir LabEx (laboratory of excellence) program, DISTALZ (Development of Innovative Strategies for a Transdisciplinary approach to Alzheimer's disease). The authors declare that there are no conflicts of interest relevant to this work.

**Financial Disclosures for the Previous 12 Months:** EM had consultancies fees from Abbvie, Biogen and Merz. The authors declare that there are no additional disclosures to report.

## Financial Disclosures and Conflicts of Interest

Author disclosures are available in the Supporting Information.

## Supporting information


**Data S1.** Details of the classification of the two missense variants according to the international classification of the American College of Medical Genetics and Genomics; supplementary references.

**Data S2.** Coi_disclosure.

**Video S1.** Age 51, on Rasagiline, Baclofen treatment and 4 h after 50 mg levodopa intake: From beginning to 33 s: lower limb dyskinesia. From 35 s to 1 min 29 s: lower limb akinesia. From 1 min 33 s to 1 min 56 s: left predominant upper limb akinesia. From 2 min to 2 min and 49 s: pyramidal stiffness and pseudo foot drop: the patient had a combined dystonia and pyramidal stiffness resulting in a pseudo foot drop.
**Video S2.** Age 63, at last follow‐up and on rasagiline, amantadine, piribedil and trihexyphenidyl medication: From beginning to 26 s: predominant spastic gait. From 27 to 55 s: predominant right hypokinesia at leg agility test. From 55 s to 1 min 14 s: predominant right hypokinesia and bilateral rest tremor (6 Hz). From 1 min 20 s to 1 min 31 s: upper limb akinesia. In all, the akinetic‐rigid syndrome involve all limbs, with right‐sided predominance.

## Data Availability

The data that support the findings of this study are available from the corresponding author upon reasonable request.
