# Supplementary material for: Expanding the Phenotype of PARK‐ PRKN to Spastic Paraplegia: A Report of Two Cases
Source: Mov Disord Clin Pract. 2026 Apr 30:10.1002/mdc3.70662. Online ahead of print. doi: 10.1002/mdc3.70662 (PMC13339402; doi:10.1002/mdc3.70662)
Supplement: Supplementary file 1 — Data S1. Details of the classification of the two missense variants according to the international classification of the American College of Medical Genetics and Genomics; supplementary references. [file MDC3-9999-0-s001.docx]

# **TITLE**

Expanding the phenotype of PARK-*PRKN* to spastic paraplegia: a report of two cases

# **SUPPLEMENTARY DATA**

**Classification of the two missense variants:**

***1) c.1298A>C, p.(His433Pro)***

The variant NM_004562.3: c.1298A>C, p.(His433Pro) in the *PRKN* gene was classified as “Likely pathogenic” according to the ACMG international criteria (1). We included criteria PM2 (Minor allele frequency in gnomAD v4 Exome = 2.054e^-06^), PM3 (*in trans* with the pathogenic deletion of exons 3 to 6 of the *PRKN* gene) and PP3 as a moderate evidence (2) using the following prediction software: BayesDel, CADD, EA, FATHMM, GERP++, MPC, MutPred2, PhyloP, PolyPhen2, PrimateAI, REVEL, SIFT, VEST4). This variant was also previously reported in the literature (3) (PP5).

***2) c.823C>T, p.(Arg275Trp)***

The variant NM_004562.3: c.823C>T, p.(Arg275Trp) was classified as “Pathogenic” according to the ACMG international criteria. We included criteria PM2 (Minor allele frequency in gnomAD v4 Exome = 0.0031), PM3 (*in trans* with the pathogenic deletion of exons 3 to 6 of the *PRKN* gene) and PP3 as a supporting evidence using the following prediction software: BayesDel, CADD, EA, FATHMM, GERP++, MPC, MutPred2, PhyloP, PolyPhen2, PrimateAI, REVEL, SIFT, VEST4). It cosegregated with disease in multiple affected family members in the databases (ClinVar IDs: 7050). This correspond to the criterion PP1 as a very strong evidence. Functional studies (PS3) demonstrated a damaging effect on the protein (4). This variant was also previously reported in the literature as pathogenic (3,5) (PP5).

**Supplementary references**

1. Richards S, Aziz N, Bale S, et al. Standards and guidelines for the interpretation of sequence variants: a joint consensus recommendation of the American College of Medical Genetics and Genomics and the Association for Molecular Pathology. Genetics in Medicine 2015;17(5):405–424. https://doi.org/10.1038/gim.2015.30

2. Pejaver V, Byrne AB, Feng B-J, et al. Calibration of computational tools for missense variant pathogenicity classification and ClinGen recommendations for PP3/BP4 criteria. The American Journal of Human Genetics 2022;109(12):2163–2177. https://doi.org/10.1016/j.ajhg.2022.10.013

3. Menon PJ, Sambin S, Criniere-Boizet B, et al. Genotype–phenotype correlation in PRKN-associated Parkinson’s disease. npj Parkinsons Dis 2024;10(1):72. https://doi.org/10.1038/s41531-024-00677-3

4. Cookson MR. RING finger 1 mutations in Parkin produce altered localization of the protein. Human Molecular Genetics 2003;12(22):2957–2965. https://doi.org/10.1093/hmg/ddg328

5. Abbas N, Lucking CB, Ricard S, et al. A Wide Variety of Mutations in the Parkin Gene Are Responsible for Autosomal Recessive Parkinsonism in Europe. Human Molecular Genetics 1999;8(4):567–574. https://doi.org/10.1093/hmg/8.4.567
