# Supplementary figures and images for: Expanding the Phenotype of PARK‐ PRKN to Spastic Paraplegia: A Report of Two Cases
Source: Mov Disord Clin Pract. 2026 Apr 30:10.1002/mdc3.70662. Online ahead of print. doi: 10.1002/mdc3.70662 (PMC13339402; doi:10.1002/mdc3.70662)

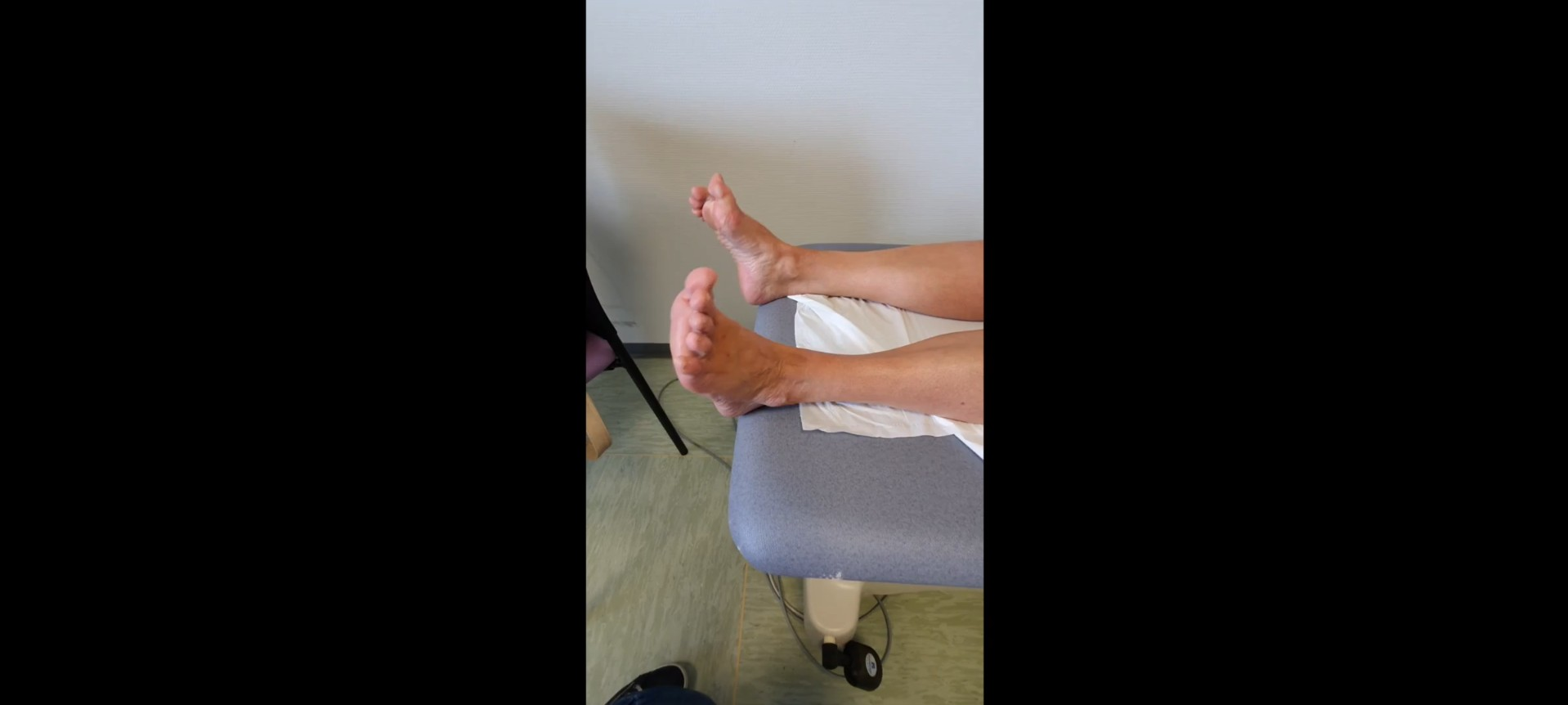

Supplement: Supplementary file 3 — Video S1. Age 51, on Rasagiline, Baclofen treatment and 4 h after 50 mg levodopa intake: From beginning to 33 s: lower limb dyskinesia. From 35 s to 1 min 29 s: lower limb akinesia. From 1 min 33 s to 1 min 56 s: left predominant upper limb akinesia. From 2 min to 2 min and 49 s: pyramidal stiffness and pseudo foot drop: the patient had a combined dystonia and pyramidal stiffness resulting in a pseudo foot drop. Video S2. Age 63, at last follow‐up and on rasagiline, amantadine, piribedil and trihexyphenidyl medication: From beginning to 26 s: predominant spastic gait. From 27 to 55 s: predominant right hypokinesia at leg agility test. From 55 s to 1 min 14 s: predominant right hypokinesia and bilateral rest tremor (6 Hz). From 1 min 20 s to 1 min 31 s: upper limb akinesia. In all, the akinetic‐rigid syndrome involve all limbs, with right‐sided predominance. [file MDC3-9999-0-s003.zip › mdc370662-sup-0002-Videos/MDC3_70662_f1_Video1_Image.tiff]
